# Genome-wide association and transcriptome studies identify candidate genes and pathways for feed conversion ratio in pigs

**DOI:** 10.1186/s12864-021-07570-w

**Published:** 2021-04-22

**Authors:** Yuanxin Miao, Quanshun Mei, Chuanke Fu, Mingxing Liao, Yan Liu, Xuewen Xu, Xinyun Li, Shuhong Zhao, Tao Xiang

**Affiliations:** 1 Key Laboratory of Agricultural Animal Genetics, Breeding and Reproduction of Ministry of Education & Key Laboratory of Swine Genetics and Breeding of Ministry of Agriculture, Huazhong Agricultural University, Wuhan, 430070 China; 2 The Cooperative Innovation Center for Sustainable Pig Production, Wuhan, 430070 China; 3 Jingchu University of Technology, Jingmen, 448000 China; 4 Agriculture and Rural Affairs Administration of Jingmen City, Jingmen, 448000 China

**Keywords:** GWAS, Transcriptomics, Feed conversion ratio, Pigs, Pathways, Hypothalamus

## Abstract

**Background:**

The feed conversion ratio (FCR) is an important productive trait that greatly affects profits in the pig industry. Elucidating the genetic mechanisms underpinning FCR may promote more efficient improvement of FCR through artificial selection. In this study, we integrated a genome-wide association study (GWAS) with transcriptome analyses of different tissues in Yorkshire pigs (YY) with the aim of identifying key genes and signalling pathways associated with FCR.

**Results:**

A total of 61 significant single nucleotide polymorphisms (SNPs) were detected by GWAS in YY. All of these SNPs were located on porcine chromosome (SSC) 5, and the covered region was considered a quantitative trait locus (QTL) region for FCR. Some genes distributed around these significant SNPs were considered as candidates for regulating FCR, including *TPH2*, *FAR2*, *IRAK3*, *YARS2*, *GRIP1*, *FRS2*, *CNOT2* and *TRHDE*. According to transcriptome analyses in the hypothalamus, *TPH2* exhibits the potential to regulate intestinal motility through serotonergic synapse and oxytocin signalling pathways. In addition, *GRIP1* may be involved in glutamatergic and GABAergic signalling pathways, which regulate FCR by affecting appetite in pigs. Moreover, *GRIP1*, *FRS2*, *CNOT2*, and *TRHDE* may regulate metabolism in various tissues through a thyroid hormone signalling pathway.

**Conclusions:**

Based on the results from GWAS and transcriptome analyses, the *TPH2*, *GRIP1*, *FRS2*, *TRHDE*, and *CNOT2* genes were considered candidate genes for regulating FCR in Yorkshire pigs. These findings improve the understanding of the genetic mechanisms of FCR and may help optimize the design of breeding schemes.

**Supplementary Information:**

The online version contains supplementary material available at 10.1186/s12864-021-07570-w.

Improving the feed conversion ratio (FCR) has become an imperative for the pig industry [1, 2]. FCR is influenced by many factors, such as metabolism, body composition and physical activity. Genetic effects must also be considered in improving FCR [1, 3, 4]. Artificial selection can effectively improve FCR, but this progress is time-consuming and expensive [5]. Therefore, elucidating the genetic mechanisms underpinning FCR and identifying genes that are significantly associated with FCR could enhance the efficiency of FCR improvement.

Genome-wide association analysis (GWAS) has previously been demonstrated as an effective method for detecting genetic variants and candidate genes associated with FCR [6–8]. Overall, a large number of single-nucleotide polymorphisms (SNPs) located on SSC 1, SSC 4, SSC 6, SSC 7 and SSC X have been identified as significantly associated with FCR. Additionally, some QTL regions and candidate genes have been reported to be associated with FCR through GWAS [9–12]. The marker WU_10.2_7_18377044 on SSC 7 has been reported to explain approximately 2.37% of phenotypic variance in residual feed intake (RFI), and DRGA0001676 on SSC 1 explained 3.22 and 5.46% of phenotypic variance in FCR and RFI, respectively [6]. Furthermore, QTL regions for RFI were detected on SSCs 1, 8, 9, 13 and 18 [8]. In addition, *MC4R*, *XIRP2*, *TTC29*, *SOGA1*, *GRK5*, *PROX1*, *NMBR*, *KCTD16*, *ASGR1*, *PRKCQ*, *PITRM1* and *TIAM1* have been reported as candidate genes for FCR in pigs by GWAS [9–12].

Transcriptome sequencing has also been comprehensively used to identify candidate genes and to elucidate the molecular mechanisms of FCR. The pathways of hormonal regulation, Notch signalling, and Wnt signalling in pituitary tissue have been reported to regulate FCR in pigs [13]. Additionally, VA metabolism, which can regulate fatty acid and steroid hormone metabolism in the liver tissue of pigs, has been found to be associated with FCR [14]. Moreover, in skeletal muscle tissue, genes involved in mitochondrial energy metabolism were downregulated, and genes involved in skeletal muscle differentiation and proliferation were upregulated, in the skeletal muscle tissues of pigs with low FCR [15]. Gradient boosting machine learning applied to muscle transcriptomes indicated that *FKBP5*, *MUM1*, *AKAP12*, *FYN*, *TMED3*, *PHKB*, *TGF*, *SOCS6*, *ILR4*, and *FRAS1* were related to FCR in pigs [16]. Transcriptomes in caecal and colonic mucosal tissues indicated that energy and lipid metabolism can affected FCR and that *GUCA2A*, *GUCA2B*, *HSP70.2*, *NOS2*, *PCK1*, *SLCs*, and *CYPs* were negatively associated with FCR in pigs [17]. Although these studies have successfully identified some important signalling pathways and candidate genes in FCR, much remains to be clarified about the molecular mechanisms of FCR.

To our knowledge, few studies have integrated the results of GWAS and transcriptome analyses to identify the major genes and crucial signalling pathways of FCR in pigs. Thus, the objectives of our study were to identify QTLs and to unravel the genetic architecture of FCR in Yorkshire pigs by performing both GWAS and transcriptome analyses in pig tissues that are related to the progress of FCR. This integrated analysis may help to enhance the power and efficiency of identifying candidate genes and key signalling pathways of FCR in Yorkshire pigs.

## Results

### Genome-wide association analyses for FCR

**Fig. 1:**
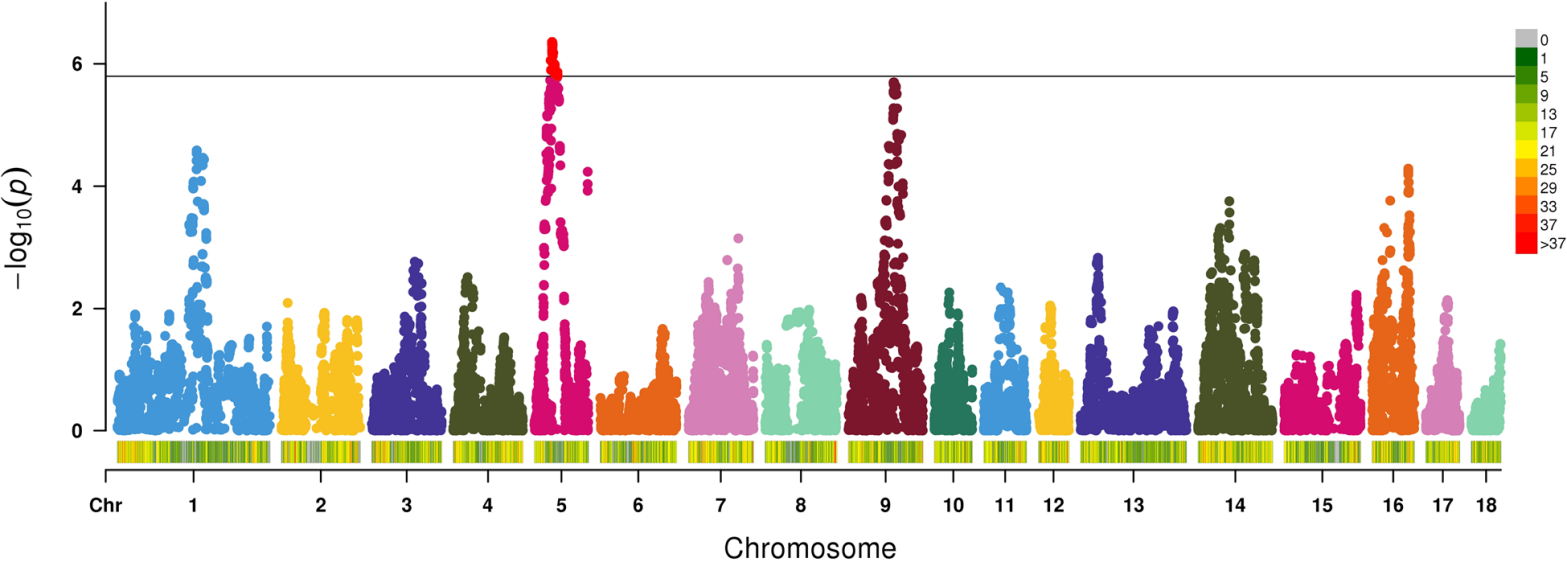
Manhattan plot of genome-wide associated analysis studies for FCR. The solid line indicates a Bonferroni corrected *p*-value = 5.796

In total, 61 SNPs reached the significance threshold of 5.796, which was calculated as the cut-off after Bonferroni correction (= − log10(0.05/31326)) (Fig. 1) [18]. All the significantly associated SNPs (61 SNPs) were located on SSC5. Among these SNPs, most (54 SNPs) are located within the region of 36.1–44.3 Mb on SSC5, while 5 SNPs are located within the region of 47.1–47.8 Mb and 2 SNPs are located within the region of 33.4 ~ 34.5 Mb.

### LD block, associated region analysis and candidate genes identified for FCR

**Fig. 2:**
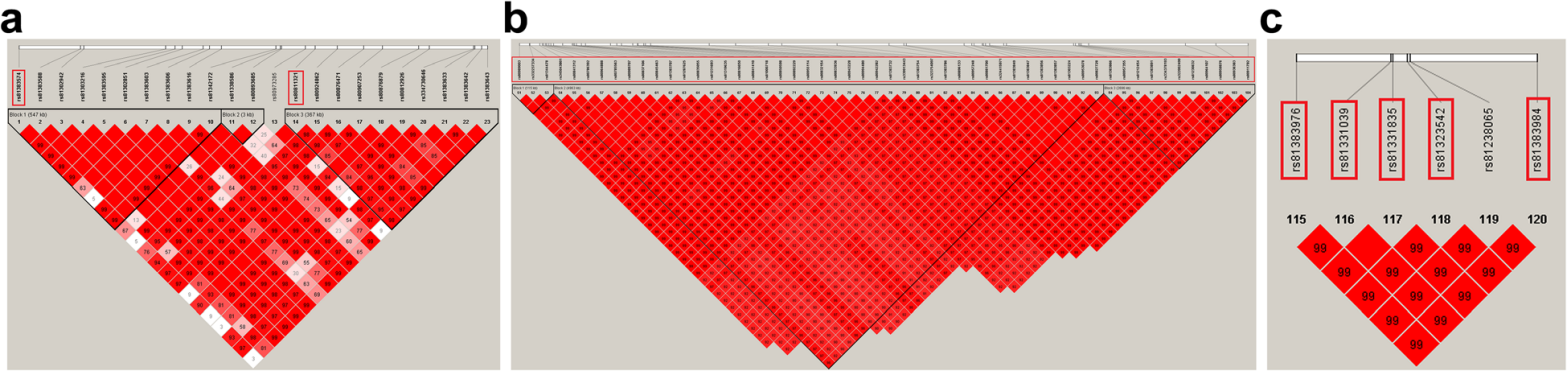
Linkage disequilibrium block on chromosome 5. Markers in the block are shown in bold. **a** Linkage disequilibrium block detected in the regions from 33.4 to 34.5 Mb on SSC5, **b** Linkage disequilibrium block detected in the regions from 36.1 to 44.3 Mb on SSC5, **c** Linkage disequilibrium block detected in the regions from 47.1 to 47.8 Mb on SSC5. SNPs in red boxes are significantly associated with FCR

Several linkage disequilibrium (LD) blocks were detected in the regions where the 61 significantly associated SNPs were located: 3 LD blocks were detected in the region of 33.4–34.5 Mb on SSC5; 3 LD blocks were detected in the region of 36.1–44.3 Mb on SSC5, and 1 LD block was detected in the region of 47.1–47.8 Mb on SSC5 (Fig. 2). The 33.4–34.5 Mb, 36.1–44.3 Mb, and 47.1–47.8 Mb regions on SSC5 maps in the Sscrofa 10.2 genome assembly were realigned to 30.2–31.3 Mb, 33.6–41.08 Mb, 43.8–44.5 Mb on SSC5 in the Sscrofa 11.1 genome assembly by NCBI Remap. Then, PigQTLdb [19] was used to identify QTLs in these regions, and the results showed that these regions contained QTLs regulating the traits of days to 110 kg, feed intake, average daily gain, body weight, loin percentage, intramuscular fat content, average backfat thickness, etc. (Table S1). Feed intake and growth traits are tightly related to the performance FCR. Thus, these regions were also considered crucial QTL regions associated with FCR.

All detailed information on the significantly associated SNPs identified by GWAS and the putative candidate genes in this QTL region is shown in Table S2. Among the 61 identified significantly associated SNPs, 26 SNPs were located within different genes. These significant SNPs, together with their corresponding genes, are shown in Table 1. Several other genes located in the 0.5 Mb genome region flanking the significantly associated SNPs were also considered candidate genes, including fibroblast growth factor receptor substrate 2 (*FRS2*), tryptophan hydroxylase 2 (*TPH2*), thyrotropin releasing hormone degrading enzyme (*TRHDE*), GLI pathogenesis related 1 (*GLIPR1*) and fatty acyl-CoA reductase 2 (*FAR2*). The ISwine platform [20] was also used to identify candidate genes for FCR in pigs. All the candidate genes identified by the ISwine platform are shown in Table S3. Based on the results from ISwine, the *TRHDE*, *TPH2*, *FAR2*, *FRS2*, and *GLIPR1* genes were confirmed as candidate genes for regulating FCR in Yorkshire pigs.

**Table 1:** Summary of within-gene significant SNPs for FCR trait

SNP ID	bp (SSC10.2)	bp (SSC11.1)	Pvalue	Genes
rs80841312	36,496,185	33,897,913	4.39E-07	*CCT2*
rs80786392	36,510,853	33,912,700	4.51E-07	*BEST3 CCT2*
rs80837106	36,589,679	33,991,092	4.51E-07	*CCT2*
rs80845463	36,621,274	34,022,700	4.51E-07	*CCT2*
rs81383707	36,721,314	34,122,773	4.54E-07	*MYRFL*
rs80964888	36,532,511	33,934,311	4.72E-07	*BEST3 CCT2*
rs332237334	36,353,885	33,842,149	4.79E-07	*FRS2*
rs81344478	36,357,722	33,838,344	4.79E-07	*FRS2*
rs80850598	37,318,776	34,747,588	4.93E-07	*PTPRB*
rs81287625	36,826,851	34,177,721	5.25E-07	*MYRFL*
rs345043801	36,469,745	33,871,482	6.03E-07	*CCT2*
rs80785563	36,544,839	33,946,621	6.03E-07	*BEST3 CCT2*
rs80989707	36,568,996	33,970,407	6.35E-07	*CCT2*
rs339913443	38,629,120	35,929,672	6.61E-07	*TPH2*
rs80835055	36,838,800	34,189,654	7.00E-07	*MYRFL*
rs81000718	37,249,647	34,677,764	7.01E-07	*PTPRB*
rs80892229	37,369,531	34,769,398	7.44E-07	*PTPRB*
rs323754097	39,138,147	36,346,640	9.75E-07	*TRHDE*
rs81383732	38,337,110	35,634,440	1.01E-06	*ZFC3H1*
rs80811321	34,095,144	30,820,701	1.26E-06	*GRIP1*
rs81323542	47,441,081	44,096,325	1.37E-06	*TMTC1*
rs81212454	42,358,084	38,794,710	1.46E-06	*GLIPR1 KRR1*
rs81383891	42,378,400	38,815,027	1.46E-06	*GLIPR1 KRR1*
rs81331039	47,398,882	44,127,767	1.49E-06	*TMTC1*
rs81331835	47,404,818	44,121,830	1.49E-06	*TMTC1*
rs81383984	47,782,626	44,464,360	1.58E-06	*FAR2*

### Integration of GWAS and transcriptome analyses

**Fig. 3:**
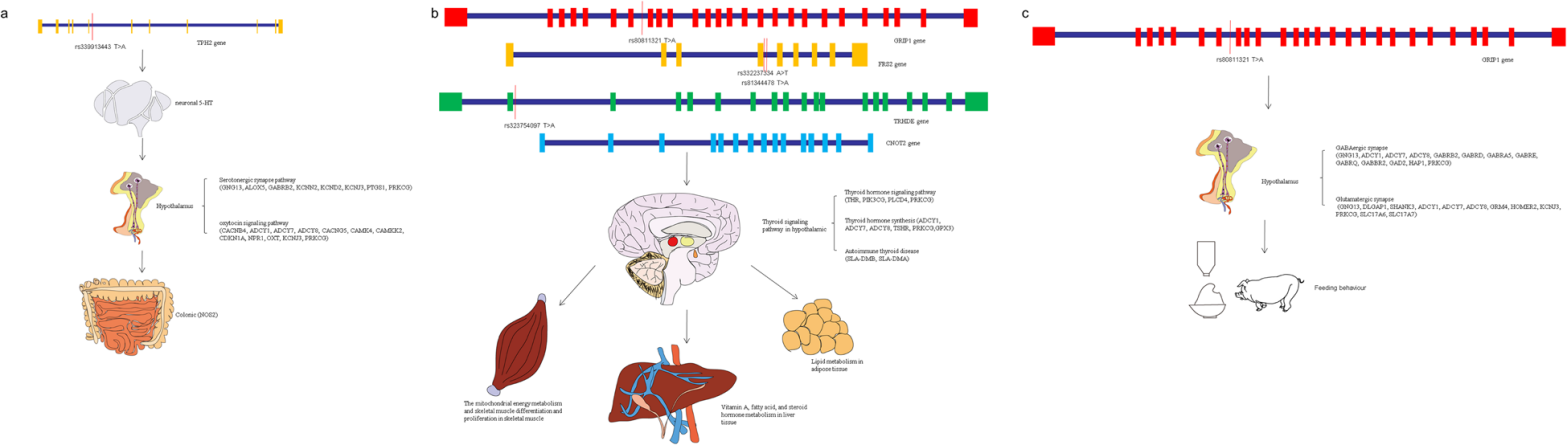
Theoretical models of the functional actions of candidate genes in modulating the feed conversion ratio. **a** *TPH2* regulates intestinal motility by serotonergic synapses and the oxytocin signalling pathway in the hypothalamus. *TPH2* produces 5-HT, and 5-HT transmits signals to oxytocin neurons through serotonergic synapses and subsequently regulates intestinal peristalsis under the action of the oxytocin signalling pathway. **b** *GRIP1*, *FRS2*, *CNOT2*, and *TRHDE* genes regulate metabolism in various tissues by the thyroid hormone signalling pathway. First, *GRIP1*, *FRS2*, *CNOT2* and *TRHDE* regulate the thyroid signalling pathway in the hypothalamus, and subsequently, the thyroid signalling pathway participates in regulating the metabolism in skeletal muscle, liver and fat. **c** *GRIP1* regulates appetite by glutamatergic and GABAergic signalling

To clarify the genetic mechanisms involved in the regulation of FCR in pigs, we integrated the GWAS results with previously published FCR transcriptome data by using DAVID [21]. The discovered signalling pathways and possible major genes are shown in Fig. 3. A mutation in the *TPH2* gene may influence the expression of the neurotransmitter serotonin (5-HT), which mediates colonic motility by the secretion of hypothalamic oxytocin (Fig. 3a, Table 2). In addition, a mutation in the *GRIP1* gene may influence the aggregation of GABA and glutamate, which mediates appetite in pigs (Fig. 3cc, Table 2). Notably, several genes involved in the regulation of thyroid hormone signalling, namely, *GRIP1*, *FRS2*, *CNOT2*, and *TRHDE*, were significantly differentially expressed in pigs with high or low FCR. The thyroid hormone signalling pathway participates in the regulation of metabolism in various tissues (Fig. 3b, Table 2). All detailed information on the differentially expressed genes identified by RNA-seq in different tissues is shown in Table S5, S6, S7 and S8.

**Table 2:** Summary of identified pathways, DEGs and genes with significant SNPs

SNP ID	Candidate Gene	Pathway			Differentially expressed genes
		Pathway name	P-Value	FDR	
rs80811321	GRIP1	GABAergic synapse	1.1E-6	2.4E-5	GNG13,ADCY1,GABRB2, ADCY8, ADCY7,GABRD,GABRE,GABRQ,GABBR2,GAD2,HAP1,PRKCG
		Glutamatergic synapse	4.0E-7	1.3E-5	GNG13,DLGAP1,SHANK3, ADCY1,ADCY8,ADCY7,GRM4, HOMER2,KCNJ3,PRKCG,SLC17A6,SLC17A7
rs339913443	TPH2	Serotonergic synapse	1.6E-3	1.2E-2	ALOX5,GABRB2,KCNN2,KCND2,KCNJ3,PTGS1,PRKCG
		Oxytocin signaling pathway	1.2E-4	1.6E-3	ADCY1,ADCY8,ADCY7,CACNG5,CAMK4,CAMKK2,CDKN1A,NPR1,OXT,KCNJ3,PRKCG,CACNB4,
rs80811321 rs332237334 rs81344478 rs323754097	GRIP1 FRS2 FRS2 TRHDE CNOT2	Thyroid hormone synthesis	1.8E-2	8.0E-2	ADCY1,ADCY8,ADCY7,GPX3,PRKCG,TSHR,
		Thyroid hormone signaling pathway	3.4E-1	6.7E-1	TRH,PIK3CG,PLCD4,PRKCG,
		Autoimmune thyroid disease	8.1E-1	8.2E-1	SLA-DMB,SLA-DMA

*CNOT2: There were no significant SNP located within the gene, but there were significant SNPs located in the upstream and downstream of the gene

## Discussion

### QTLs, LD blocks and candidate genes for FCR

Feed efficiency (FE) is an important economic trait that greatly affects the economic profit of the breeding industry. The identification of major genes regulating FE may help to enhance the efficiency of improving FE through molecular breeding technology. However, only a few candidate genes have been identified due to the difficulty of analysing ratio traits and to sample size limitation. In the current study, we implemented a genome-wide association analysis for FCR in a large Yorkshire population. Our analyses identified a series of novel significant SNPs located in the 33.4–34.5 Mb, 36.1–44.3 Mb, and 47.1–47.8 Mb regions on SSC5. LD analysis showed that these regions are highly linked, and many QTLs related to feed intake and growth traits are located in these regions. Logically, these regions were considered candidate QTL regions for FCR. Genes located within 1 Mb of the significantly associated SNPs, including fatty acyl CoA reductase 2 (*FAR2*), interleukin-1 receptor-associated kinase-3 (*IRAK3*), and tyrosyl-tRNA synthetase 2 (*YARS2*), were inferred as candidate genes regulating FCR in our study.

The *FAR2* gene spans 44.38 Mb to 44.55 Mb on SSC5. It is a key gene for fatty acid β-oxidation, acetyl-CoA translocation, peroxisome biogenesis, and the glyoxylate cycle [22]. Moreover, *FAR2* was associated with insulin resistance [23]. Previous studies reported that lipid metabolism can explain the variation in FCR [14, 24, 25]. Therefore, the *FAR2* gene might be a candidate gene for FCR.

*IRAK3* belongs to the serine-threonine kinases family and is negatively correlated with the mitochondrial oxidative stress marker *SOD2*. It has been reported that high *IRAK3* and low *SOD2* cause weight loss [26, 27]. Previous studies reported that decreased *IRAK3* was associated with increased mitochondrial reactive oxygen species (ROS) [28], and other studies have reported that ROS can decrease muscle mass by regulating mitochondrial biogenesis and the expression of antioxidant genes [29, 30]. Mitochondrial energy metabolism is a factor potentially affecting the feed conversion ratio in pigs [15]. Therefore, *IRAK3* is worthy of further functional investigation.

*YARS2* is a key factor that binds tyrosine to its corresponding mt-tRNA for the synthesis of mitochondrial proteins. Mutations in *YARS2* can lead to mitochondrial respiratory chain complex deficiencies and are related to mitochondrial myopathy [31, 32]. *YARS2* has not been functionally characterized in pigs. However, since its function involves mitochondrial protein synthesis and mitochondrial respiration, it might be an important candidate gene for FCR in pigs.

### *GRIP1* controls appetite through glutamatergic and GABAergic signalling

**Fig. 4:**
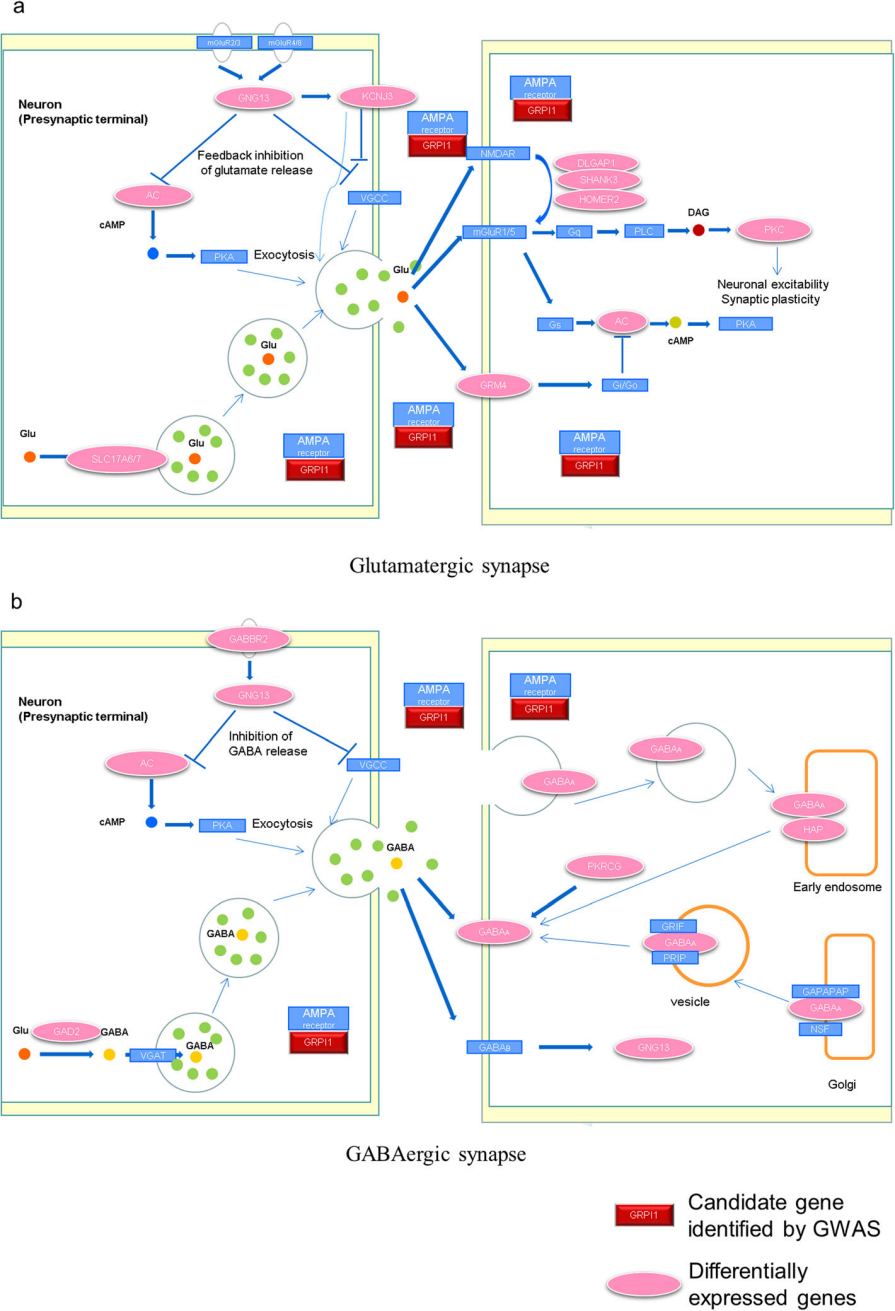
*GRIP1* regulates appetite through glutamatergic synapses (**a**) and GABAergic synapses (**b**)

In this study, we integrated GWAS results with transcriptome analyses, aiming at identify candidate genes and biological pathways associated with FCR in pigs. Feed intake is a major physiological process associated with variations in FCR [33–35]. GABA (γ-amino-butyric acid) and glutamate, which are expressed in hypothalamic neurons, can promote feeding and weight gain, while *GRIP1* can interact with the C termini of AMPA receptors and cluster at both glutamatergic and GABAergic synapses [36–38]. In addition, genes associated with GABAergic synapse (*GNG13*, *ADCY1*, *ADCY7*, *ADCY8*, *GABRB2*, *GABRD*, *GABRA5*, *GABRE*, *GABRQ*, *GABBR2*, *GAD2*, *HAP1*, *PRKCG*) and glutamatergic synapse (*DLGAP1*, *SHANK3*, *ADCY1*, *ADCY7*,*ADCY8*, *GNG13*, *GRM4*, *HOMER2*, *KCNJ3*, *PRKCG*, *SLC17A6*, *SLC17A7*) were differentially expressed in hypothalamic tissue in pigs with high or low FCR performance [39]. Therefore, *GRIP1* may control appetite through glutamatergic (Fig. 4a) and GABAergic signalling pathways (Fig. 4b). Moreover, two informative SNPs in *GRIP1* were identified to be significantly associated with backfat thickness in pigs [40]. Therefore, *GRIP1* may be an important candidate gene for FCR in pigs.

### *TPH2* may affect 5-HT secretion, thereby mediating intestinal motility through the hypothalamic oxytocin signalling pathway

**Fig. 5:**
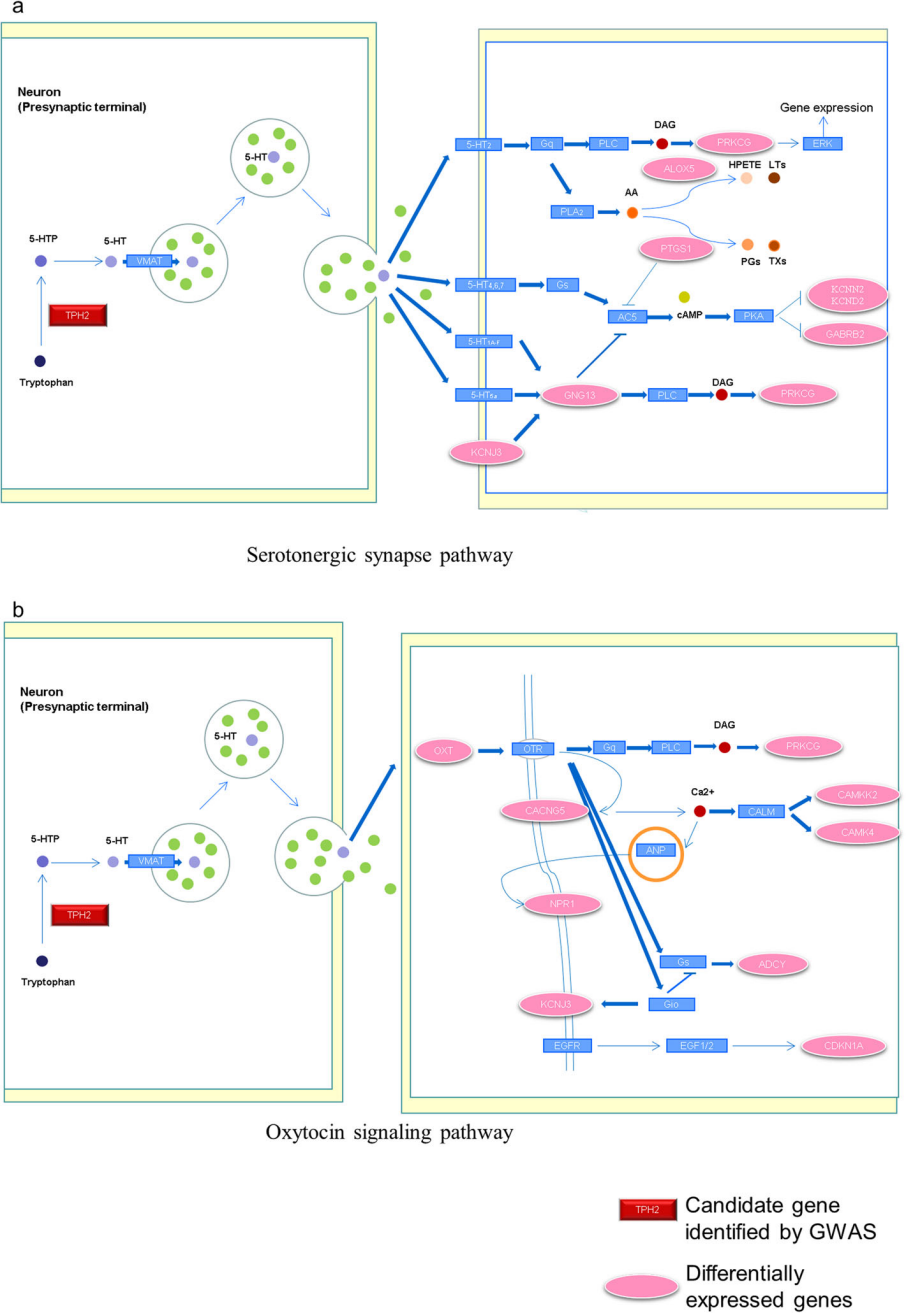
5-HT, produced by *TPH2*, regulates the serotonergic synapse pathway (**a**) and oxytocin signalling pathway (**b**) in the hypothalamus

Brain-gut interactions may be an important factor in FCR in pigs [41]. The central neurotransmitter serotonin (5-hydroxytryptamine, 5-HT), produced by tryptophan hydroxylase 2 (*Tph2*), mediates colonic motility by regulating oxytocin (OT) synthesis in the hypothalamus [42, 43]. In addition, knockout of *TPH2* in mice resulted in depletion of 5-HT in the brain, and the mice showed increased food consumption [44]. Therefore, *TPH2* can regulate appetite and intestinal motility by affecting the secretion of 5-HT. In our results, a significant SNP (rs339913443) was found to be located in the *TPH2* gene. Moreover, transcriptome sequencing in the hypothalamus of pigs with extremely high or low feed efficiency revealed that genes related to serotonergic synapse (*GNG13*, *ALOX5*, *GABRB2*, *KCNN2*, *KCND2*, *KCNJ3*, *PTGS1*, *PRKCG*) (Fig. 5a) and the oxytocin signalling pathway (*ADCY1*, *ADCY7*, *ADCY8*, *CACNG5*, *CAMK4*, *CDKN1A*, *CACNB4*, *CAMKK2*, *NPR1*, *OXT*, *KCNJ3*, *PRKCG*) (Fig. 5b) were differentially expressed [39]. RNA-seq in the caecal and colonic mucosa revealed that *NOS2*, which is related to gastrointestinal peristalsis, was a candidate gene for FCR [17]. Therefore, the SNPs within *TPH2* may change the expression of this gene, thereby affecting the secretion of 5-HT. Sequentially, 5-HT regulates intestinal motility through the oxytocin signalling pathway in the hypothalamus.

### *GRIP1*, *FRS2*, *CNOT2*, *TRHDE* may affect metabolic processes

Thyroid hormone (TH), which is regulated by thyrotropin releasing hormone (TRH) and thyroid stimulating hormone (TSH), is in turn involved in regulating many metabolic processes essential for growth and development, including basal metabolic rate, facultative thermogenesis, skeletal muscle growth, regulation of body weight, and lipid metabolism [45–47]. Thyroid hormone receptors (TRs) mediate the biological effects of thyroid hormone (T3) [48]. In our study, many candidate genes participated in regulating TH signalling, including *GRIP1*, *FRS2*, *CNOT2*, and *TRHDE*. Among them, *GRIP1* acts as a coactivator for TR, strengthening the combination of TR and TH [49, 50]. *FRS2* is involved in FGF21-AMPK signalling and can be induced to accelerate energy metabolism through thyroid hormone [51]. *CNOT2* is an important regulator of energy metabolism, cellular stress and fatty acid metabolism in skeletal muscles. The heterozygous intragenic deletion of *CNOT2* displayed disordered phenotypes including learning disabilities, developmental delays, and hypothyroidism [52, 53]. *TRHDE* is an extracellular peptidase that specifically degrades *TRH* to regulate appetite and metabolism [54, 55]. SNP association analysis in a new Ujumqin Sheep population showed that the *TRHDE* gene was significantly associated with body weight [56]. Moreover, transcriptome sequencing in the hypothalamus of pigs with high or low FCR revealed that genes involved in the thyroid hormone signalling pathway (*TRH*, *PIK3CG*, *PLCD4*, *PRKCG*), thyroid hormone synthesis (*ADCY1*, *ADCY7*, *ADCY8*, *GPX3*, *PRKCG*, *TSHR*) and autoimmune thyroid disease (SLA-DMB, SLA-DMA) were differentially expressed [39]. Therefore, differences of FCR in pigs were mediated by the thyroid signalling pathway in the hypothalamus, leading to different phenotypes and differential gene expression in muscle, fat, liver and others tissues.

Overall, this study used GWAS to discover SNPs significantly associated with FCR. None of the significant sites changed the corresponding protein coding, but this study found that the downstream genes in the pathway were significantly differentially expressed in the high-FE and low-FE groups. These results indicate that the SNPs found in this study may play a regulatory role. However, this possibility needs to be verified with other omics data in the future. To further confirm the causal genes, integration analyses of GWAS and eQTLs should be implemented.

## Conclusions

The present study detected a novel QTL region on SSC5 that is significantly associated with FCR in Yorkshire pigs. An integrative analysis of the GWAS results and transcriptome results in different tissues has been used to identify candidate genes and signalling pathways that play a decisive role in this trait. *GRIP1*, *TPH2*, *FRS2*, *CNOT2*, and *TRHDE* were suggested to be the most likely candidate genes for FCR. These findings offer a better understanding of the molecular mechanisms regulating FCR in pigs.

## Materials and methods

### Phenotype recordings

In this study, all FCRs (feed intake/weight gain) were measured at intervals of 30 to 100 kg in Yorkshire pigs by a pig performance testing system in a national pig nucleus herd. In total, FCR recordings were collected from 14,401 pigs. All of the phenotypic recordings were measured between 2017 and 2020. Pedigrees can be traced back for ten generations. In total, 19,811 pigs are included in the pedigree. Genomic selection was started later in 2018, and since then ear tissues were collected following a criteria that at least 2 males and 2 females in each litter should be collected. As a result, 3672 YY pigs contained both FCR recordings and ear tissue samples. All experimental protocols were approved by the Ethics Committee of Huazhong Agricultural University (HZAUMU2013–0005).

### Genotypes

The SNP markers were genotyped in 3672 YY pigs by using an Illumina PorcineSNP60 Genotyping BeadChip. SNPs were mapped to pig chromosomes using Sscrofa genome build 10.2 [57]. Quality controls were applied as follows: Samples with call rates lower than 90% were removed, SNPs with call rates smaller than 90% were removed, and SNPs with minor allele frequencies smaller than 0.05 were filtered out; SNPs that deviated strongly from Hardy-Weinberg equilibrium within breeds (*p* < 10^− 7^) were also excluded. After quality control, 3672 YY pigs and 31,236 SNPs distributed over the 18 porcine autosomes were used for genome-wide association analysis.

### Statistical model for the prediction of genomic breeding values

The single-step GBLUP (ssGBLUP) method was used to predict genomic breeding values (GEBVs) [58, 59]:

1 $$ \mathbf{y}=\mathbf{Xb}+\mathbf{Zu}+\mathbf{e} $$

where y contained phenotypic recordings for FCR; **Xb** indicated the fixed effects, including unit-year-month effect, sex effect and covariate for the starting weight; **u** was random additive effect and **Z** was the incidence matrix to relate the additive effects to the phenotypic recordings; and **e** was a vector of residual effects. It was assumed that the random additive effects followed a normal distribution, as follows: $\mathbf{u}\sim N\left(0,\boldsymbol{H}{\sigma}_u^2\right),$ where ***H*** was the combined pedigree and genomic information relationship matrix [58].

To remove the contribution of information from relatives, de-regressed estimated breeding values (DEBVs) were used as the response variable in GWAS analysis [60], which can be calculated by weighting EBVs [61]. The weighting factor (*w*_*i*_) for animal i can be calculated as follows:

2 $$ {w}_i=\frac{1-{h}^2}{\left(c+\left[\left(1-{r}_i^2\right)/\left({r}_i^2\right)\right]\right){h}^2} $$

where *h*^2^ is the heritability of the trait, ${r}_i^2$ is the reliability of EBV of the ith animal, and c is the proportion of genetic variation that could not be explained by the genetic information. In this study, c was assumed to be constant at 0.5 [62].

### Genome-wide association studies

The genome-wide association study was performed on 3672 genotyped pigs by using the MLMA (mixed linear model-based association analysis) option in GCTA software [63]. All SNPs were used for the association analysis. The mixed linear model was:

3 $$ \mathbf{y}=\mathbf{1}\upmu +\mathbf{xb}+\mathbf{wg}+\mathbf{e} $$

where ***y*** was the vector of DEBVs for FCR in the genotyped Yorkshire pigs; μ was the overall mean and **1** was a vector of ones; **x** was a vector of SNP genotypes, with entries 0, 1, 2 for genotypes AA, AB and BB, respectively; b was the fixed additive genetic effect of analyzed SNP; and **g** was a vector of random polygenic effects and **w** was the incidence matrix relating the DEBVs to the corresponding random polygenic effects; It was assumed that **g** followed a normal distribution with mean of 0 and variance of $\boldsymbol{A}{\sigma}_g^2$, where ***A*** was the pedigree-based additive relationship matrix. **e** is a vector of residual effects, following a normal distribution as $\mathbf{e}\sim N\ \left(0,\boldsymbol{D}{\sigma}_e^2\right)$, where ***D*** is a diagonal matrix with elements ${d}_{ii}=\left(1-{r}_{DEBV}^2\right)/{r}_{DEBV}^2$ and ${r}_{DEBV}^2$ is the reliability of the DEBVs. A significant test of SNP effects was implemented by a two-sided t-test. Bonferroni corrections were set for the genome-wide significance threshold (−log10[0.05/number of SNPs] = 5.796).

### Detection of LD block and QTL analysis

Significant SNPs located within 1 Mb from each other were considered to belong to the same QTL region. Detection of LD blocks was performed in the chromosomal regions containing the identified significantly associated SNPs by Haploview software [64]. NCBI Remap was used to transfer the significant regions on SSC5 aligned to the Sscrofa 10.2 genome assembly to those aligned to the Sscrofa 11.1 genome assembly, since the original genomic information was mapped to SusScrofa 10.2 reference genome by the gene-sequenceing company. Then, QTLs located in these significant regions were identified by searching a pig QTL database (pigQTLdb, https://www.animalgenome.org/cgi-bin/QTLdb/SS/index).

### Candidate gene search and integrated analysis with transcriptome data

Genes that are located in the identified QTL region and the 0.5 Mb flanking these loci were considered candidate genes for regulating FCR [8, 65]. Then we used an omics knowledgebase, ISwine (http://iswine.iomics.pro), to search for candidate genes based on genome, transcriptome, quantitative traits and annotation information [20]. Transcriptome analyses in different tissues (muscle, liver, fat, hypothalamus) collected among 238 Yorkshire boars that with high or low FCR performances. These Yorkshire boars were castrated and fed in the fattening farms grown from 30 to 90 kg and the sample collection were performed in previous studies in our laboratory [14, 24, 39, 66]. Three high-FE pigs and three low-FE pigs that significantly differed were selected (*p* < 0.05) for RNA-seq. The detail information about the pigs used in RNA-seq was shown in Table S4. Total RNA was extracted from frozen muscle, fat, liver and hypothalamic tissues by using TRIzol reagent (Invitrogen, USA) and sent to Genergy Biotechnology (Shanghai, China) for library construction. Six RNA-seq libraries of muscle, fat, and liver tissues were constructed and applied for RNA-seq. However, one of the high-FE samples failed to construct a library in hypothalamic tissues, and five RNA-seq libraries (two high-FE libraries and three low-FE libraries) in hypothalamic tissues were used for RNA-seq. Salmon (version 1.4.0) was used to align sequencing reads to the pig reference genome Sscrofa 11.1 and for wicked-fast transcript quantification [67]. The negative binomial generalized liner models of DEseq2 was used to identify differentially expressed genes according to |log2FoldChange| (|log2FC|) ≥ 1 and *p*-value < 0.05. Subsequently, the genes identified by ISwine and GWAS were integrated and analysed with the transcriptome results. Database for Annotation, Visualization and Integrated Discovery (DAVID) software (https://david.ncifcrf.gov/) was used for functional classification and pathway analysis for all the identified genes [68]. The differentially expressed genes identified by RNA-seq and the genes located in the QTL region associated with FCR were input into the DAVID software to found the genes located in the same signaling pathway.

## Supplementary Information

**Additional file 1: Table S1**. Description of quantitative trait loci (QTL) in the regions significantly associated with FCR.

**Additional file 2: Table S2**. Summary information of significant SNPs and candidate genes for the FCR trait.

**Additional file 3: Table S3**. Candidate genes for FCR in pigs identified by the ISwine website.

**Additional file 4: Table S4**. Animal performance of Yorhshire pigs used in RNA sequencing.

**Additional file 5: Table S5**. List of hypothalamus DEGs between Yorkshire pigs with high and low FCR.

**Additional file 6: Table S6**. List of liver DEGs between Yorkshire pigs with high and low FCR.

**Additional file 7: Table S7**. List of muscle DEGs between Yorkshire pigs with high and low FCR.

**Additional file 8: Table S8**. List of fat DEGs between Yorkshire pigs with high and low FCR.

## Data Availability

The datasets analyzed during the current study are available from the corresponding author upon request.

## Abbreviations

CNOT2: CCR4-NOT transcription complex subunit 2
DEBV: de-regressed estimated breeding values
FAR2: fatty acyl-CoA reductase 2
FCR: Feed conversion ratio
FRS2: fibroblast growth factor receptor substrate 2
GEBVs: genomic estimated breeding values
GRIP1: GLI pathogenesis related 1
GWAS: genome-wide association study
IRAK3: interleukin 1 receptor associated kinase 3
LD block: linkage disequilibrium block
MLMA: mixed linear model based association analysis
pigQTLdb: pig QTL database
QTL: quantitative trait locus: TIAM1
SNP: single nucleotide polymorphism
ssGBLUP: single-step genotype best linear unbiased prediction
TPH2: tryptophan hydroxylase 2
TRHDE: thyrotropin releasing hormone degrading enzyme
YARS2: tyrosyl-tRNA synthetase 2
YY: Yorkshire pigs
